# Shortages Without Frontiers: Antimicrobial Drug and Vaccine Shortages Impact Far Beyond the Individual!

**DOI:** 10.3389/fmed.2021.593712

**Published:** 2021-02-12

**Authors:** Guillaume Beraud

**Affiliations:** Infectious Diseases Department, University Hospital of Poitiers, Poitiers, France

**Keywords:** antimicrobial, vaccine, shortage, out-of-stock, antimicrobial resistance, vaccine hesitancy

## Abstract

Among increasingly common drug shortages, antimicrobial drug and vaccine shortages are the most frequently reported. This could be related to the smaller size of the market, compared to statins or antidepressant drugs. But there are multiple causes to shortages, such as flawed manufacturing processes, modification of quality control processes and scarcity of raw materials. Besides, concentration of manufacturing in emerging economies, dependence on a single producer and pressure on profit margins amplify the consequences of any manufacturing problem. Antimicrobial drug shortages have an impact on patient outcomes and antimicrobial resistance (AMR) by leading to choices of alternatives with an inadequately large spectrum, and consequently with deleterious side effects and increased costs. Moreover, vaccine shortages result in controversies exacerbated by the antivax community. Given the transmissibility of infectious diseases, antimicrobial drug and vaccine shortages will impact both individual and population health through herd effect. For these reasons, they represent a worldwide threat that goes beyond impact at the individual level. There has been no coordinated response to this threat hitherto. In order to provide an adequate response plan, precise data on shortage occurrences and their causes are a prerequisite. Moreover, efficient action will not be possible without a transnational will. Examples of useful actions could be: Incorporating a standardized survey into the WHO surveillance programs on antimicrobial use; creating a communication channel between pharmaceutical companies, providers and national agencies so as to recognize upcoming shortages; licensing some laboratories to manufacture out-of-stock drugs, for the duration of the shortage.

## Introduction

Antimicrobial drug and vaccine shortages have been increasingly reported worldwide. Multiple reasons have been put forward, quality issues and narrow profit margins among others, suggesting that drug shortages are going to become more and more common. Surveys performed by hospital pharmacists in Europe (1), Australia (2), and France (3) have shown that antimicrobial shortages are predominant among medicine shortages. However, none of these studies were specifically focused on antimicrobials or vaccines.

Antimicrobial drugs and vaccines are the only medicines designed not for humans but pathogens, to prevent or cure human infection. Transmission being specific to infectious diseases, any medicine with an influence on infectious diseases will affect not only the individual using them, but the whole community. As antimicrobial drugs and vaccines are completely different from other drugs, shortages cannot be regarded as similar, insofar as they present specificities in both causes and consequences. Consequently, they require implementation of specific policies, whether to prevent such shortages or to limit their impact on individuals and the healthcare system (4). For that reason, we aim at highlighting the specificities regarding causes, consequences and challenges related to antimicrobial drug and vaccine shortages.

## Issue Extent

### The Scale of Antimicrobial Drug and Vaccine Shortages

Although both are worryingly increasing, it is necessary to distinguish common short supplies from major shortages. Indeed, a shortage of a product does not necessarily imply shortages of all products with the same international non-proprietary name. Short supplies are exceedingly frequent and concern a drug for which hospital pharmacists are usually able to find an equivalent product from another company. While they represent a considerable burden of work for the hospital pharmacist, most physicians are not aware of it, and consequences for the patient are relatively limited: An example would be the use of an Italian alternative (Sigmacillina) during the shortage of Extencilline (Benzathine benzylpenicillin), which required 2—painful—injections instead of one. On the other hand, shortages, particularly for antimicrobials and vaccines, result in complete unavailability of a necessary product, usually at the national level, and they entail consequences for both the physician and the patient. While many studies have shown an increasing trend in drug shortages, and demonstrated that antimicrobial drugs and vaccines are the most impacted, there is limited information allowing to precisely scale the extent of the phenomenon. Moreover, most of the studies have been conducted from a hospital pharmacist's point of view, with little or limited assessment of the clinical impact of the shortage (if it exists).

Compared to other drugs, antimicrobial shortages were those most commonly reported (>50%) in a 2014 survey by the European Association of Hospital Pharmacists (EAHP) on medicine supply shortages in the hospital sector that was confirmed in the 2019 EAHP survey (1, 5). Antimicrobial shortages were also the most widely reported in a survey by the Society of Hospital Pharmacists of Australia, as well as the most widely reported of all unique drug product shortages (2). Similar results were observed in a recent survey in France (3) with a trend toward an increasing number of antimicrobial shortages over recent years.

Using a US nationwide database, Quadri et al. estimated a median of 10 new antibacterial drug shortages per year between 2001 and 2013 with a sizeable rise since 2007 and median duration exceeding 6 months (6). Noticeably, a considerable proportion of shortages were without clear reasons. Using the same US database and focusing on vaccines, Ziesenitz et al. showed that even though vaccine shortages were a smaller proportion of all drugs shortages, half of them concerned the pediatric schedule, therefore resulting in vaccine deferral (7). Comparisons between countries are difficult because of different definitions of shortages, but a similar trend toward an increase has appeared in other countries (5). For many of them, national agencies report shortages and may suggest alternative strategies, but no publication has thoroughly assessed the global extent of antimicrobial drug and vaccine shortages, which consequently limits responses at the national and local level (8). Very limited information is available from low and middle-income countries (LMIC), although some reports have suggested a considerably worsening situation (4). Increasing demand for antimicrobials and vaccines has been coming from LMIC, with the highest rate of antimicrobial resistance (AMR) and a limited ability to fund policies to prevent shortages. Ironically, a large proportion of antimicrobials are produced in plants based in China and India, even though these countries are probably heavily impacted by shortages.

### Identifying Causes

While causes for antimicrobial drug and vaccine shortages are multiple and complex, they are mainly the symptoms of weak supply chains. Because there are very few competitors at the different stages of the chain, a complication at only one stage can result in a collapse of the entire chain. Because antimicrobial drugs and vaccines are less profitable than other medicines (prices are low, they are taken in short courses, particularly for vaccines), the antimicrobial drug and vaccine market has been abandoned by many pharmaceutical companies, including the largest ones, leading to reliance on a small number of manufacturers. In addition, the latter have had to rely on even fewer active pharmaceutical ingredient (API) manufacturers, most of whom are based in India and China, where manufacturing costs are lower. Of note, according to a WHO report 10 of the most commonly used antibiotics are produced by only two API manufacturers (9). As a result, a failure in one API manufacturer can lead to shortages lasting months or years. As an example, an explosion at a Chinese factory led to a prolonged and global shortage of piperacillin-tazobactam, as it was the only producer of the needed API. Furthermore, as a new antibiotic is systematically used sparingly so as to limit its impact on AMR, there is little incentive toward innovation and investment in R&D to develop new antibiotics. And if an increasing number of pharmaceutical companies leave the antimicrobial market, API manufacturers are correspondingly likely to do so.

In addition, fragile supply chains are not able to adapt to external crises leading to increased demand. A recent example is the impact of COVID-19 on BCG vaccines at the origin of a shortage due to premature expectations with regard to COVID-19 (10). But even more worrying is the fact that global antibiotic consumption increased by 65% between 2000 and 2015 (11), while projections suggest a 200% increase between 2015 and 2030. This is the result of both increasing AMR and limited implementation of antimicrobial stewardship policies. Increasing AMR leads to an increase in wide-spectrum antimicrobial consumption, which will certainly create tensions in the antimicrobial market between high income countries (which negotiate market access) and LMICs (with a huge demand and where manufacturers are located). Besides, countries with large potential markets focus their tendering processes on prices, leading pharmaceutical companies to prioritize the most profitable market over smaller markets.

When shortages are analyzed from an economistic approach, a study showed that a decrease in reimbursement was associated with a rise in shortages, as the manufacturer has to lower his prices and is therefore lacking in incentives to invest in capacity production (12). It is also unsurprising that studies have found that the drugs most impacted by shortages are the oldest and generic drugs, which offer only narrow profit margins (13). As antimicrobial drugs and vaccines are medicines affording a low profit margin, there is a limited innovative investment in this field, which means that many drugs are off-patent and susceptible to shortages, which creates a vicious circle toward less and less investment.

### Consequences and Impact

Because antimicrobial drugs and vaccines are different from other drugs as they not only impact the individual using them but the whole community through pathogens transmission, shortages have consequences that go beyond the individual. In addition to the nuisance they represent in everyday medical practice, antimicrobial shortages may have an impact on patient outcomes and AMR by leading to a choice of alternatives with an inadequately large spectrum, and result in deleterious side effects and increased costs (4, 14, 15). Indeed, replacing a narrow-spectrum, old and cheap antimicrobial by a broader spectrum, recent, and expensive one is exactly the contrary of the de-escalation process supported by antimicrobial stewardship guidelines. Focusing on antimicrobials and vaccines, Gundlapalli et al. showed that more than half of the surveyed US physicians reported adverse effects related to shortages: use of more toxic antimicrobials, more expensive agents, broader-spectrum antimicrobials, long-term morbidity from inadequate treatment of infection, and longer hospitalizations (16). The same survey was carried out 5 years later, showing that three-quarters of the respondents reported that shortages affected patient care or outcomes (17). Mention was made of the use of broader-spectrum antimicrobials than would have been optimal, of more costly agents, of second-line/less-effective therapy, of more toxic antimicrobials than would have been optimal, delayed treatment, longer hospitalization, slower clinical response, use of compounded agents, long-term morbidity from inadequate treatment of infection, patients transfer to other facilities, contracted diseases that an out-of-stock agent should have prevented.

While shortages result from a low profit and instable market, they also occasion increased costs for healthcare systems, at least for 20–30 million euros per shortage according to the WHO (18), and associated costs related to the purchase of more expensive products, loss of revenue etc. may multiply these costs by 10 (19).

Besides impacting both individual and population health through infectious transmission, vaccine shortages result in controversies exacerbated by the anti-vax community. In 2019, a suspension of meningococcal vaccine production and distribution in India, the second-largest contributor to Hajj mass gatherings called into question the optimal strategy to avoid any disruption in mandatory vaccination before entering Saudi Arabia. This type of incident runs the risk of reinforcing conspiracy theories and distrust in the healthcare systems, as has been the case for polio vaccination among Muslims in particular and also internationally (20). Furthermore, alternatives for stocked-out vaccine usually include more valences. Although there is no real evidence that it could cause adverse effects for the patient, this results in an increased cost and increased defiance toward vaccines, which in turn may decrease vaccine coverage and consequently herd immunity. Since 2013 BCG vaccine availability has been problematic in many countries, more specifically in LMICs, and interruption of BCG vaccine supply is suspected to be the cause of the resurgence of tuberculous meningitis and an upcoming peak in child mortality (21). In addition, BCG shortages related to COVID-19 crisis necessitate more aggressive treatment of bladder cancer, imposing surgery in cases where BCG vaccine would have been sufficient. Besides, the 2018 yellow fever outbreak in Brazil created an unusually large demand for tourist vaccination and a subsequent global shortage, which could be the trigger for yellow fever in non-endemic countries (22, 23). Finally, the vaccine market is an extreme version of the antimicrobial market. Largely supported vaccination campaigns originating from charity organizations have led to a relatively low price for vaccines, allowing profits practically only for non-R&D based companies, whereas R&D-based companies, on the other hand, can't sustain profitable R&D activity. As a consequence, there are hardly three providers at max for most vaccines, which renders them particularly susceptible to shortages (24).

## Proposals for Solutions and a Call for Action

Considering that you can't improve or even manage what you can't measure, the first step toward improvement is making a precise assessment of antimicrobial drug and vaccine shortage. This means not only performing surveys among pharmacists or physicians, but also exhaustively quantifying stockout and shortages, their causes and their impact on the healthcare system and patients alike.

In many countries, registries have been created where pharmaceutical companies can report upcoming shortages and their expected duration. Such a report is in many cases supposed to be mandatory, and is often cited in scientific literature as a tool to assess shortage and subsequently obtain an optimal response from the government. However, experience has shown that such tools are less than optimal insofar as shortages are often declared posteriori or when currently occurring. In 2012, the US Food and Drug Administration requested manufacturers to declare impending drug shortages in advance. A 2016 survey mentioned that 87% of the ID physicians participating did not perceive any improvement in communications about shortages since publication of the 2012 FDA requirements. A similar observation has been made in Europe (25). Indeed, appropriate communication tools need to be implemented as a complement to collection of good data. In addition, timely and precise measurement of shortages is necessary to adapt and provide a quick response that could or prevent the upcoming shortages or at least limit their consequences. But communication should go both ways. Providing precise information on antimicrobial and vaccine consumption would help to anticipate and prevent shortages. For that purpose, incorporation of a standardized survey in the WHO surveillance programs on antimicrobial use would help to collect data on both consumption and shortages. Using electronic prescribing with a computer decision support system would also help to warn the physician on a current or upcoming shortage, thereby providing an alternative to the out-of-stock drug and collecting data on antimicrobial and vaccine use. All told, early information and tools for timely response are key to managing shortages. A recent study on risk reduction related to shortages suggests that an appropriate IT system would considerably decrease hazard risk related to antimicrobial shortages (26). However, this presupposes availability and efficiency of an IT system, which may be problematic in LMICs. As an example, a survey conducted among public sector hospitals in South Africa showed that most (83.3%) of them experienced antimicrobial shortages and that less than half (42.4%) had a therapeutic interchange policy (of which only 30.2% were documented) (27). Nonetheless, in collaboration with the prescriber, the pharmacists in this study usually substituted the out-of-stock prescribed antimicrobial with an alternative, which was not the case according to a US study mentioning that 67% of substitutions were automatically performed by a pharmacist committee (28).

Another step toward improvement is to consider antimicrobial drugs and vaccines as essential medicines, taking into account their impact beyond the individual. For that purpose, the WHO list of essential medicines which categorizes antibiotics in three groups, namely Access, Watch, and Reserve should be used:

- *Access*: First- and second-line ATB that should be widely available (e.g., amoxicillin). Vaccines could be added to this category.
- *Watch*: Second-line treatments reserved for specific indications due to high risk of resistance (e.g., fluoroquinolones).
- *Reserve*: Third-line (last-resort treatment such as colistin or aztreonam, which should be dedicated to severe cases to limit resistance).

Unsurprisingly, the *Access* group concerns older antimicrobials, which are the most frequently out-of-stock, even though they should always be available at an affordable cost as they are first-line medications. These categories could be used as a model to tailor strategies according to the importance given to a specific drug. For *Access* and *Watch* categories, active collaboration between governments and pharmaceutical companies should reinforce supply chains and limit the risk of shortages. The WHO nonetheless advises that *Reserve* antibiotics should be used only when other options have failed, which requires tight control both in stewardship (to limit excessive use) and in production (to guarantee uninterrupted supply). Subsequently, collection and sharing of data on infectious disease epidemiology, antimicrobial and vaccine consumption and impending stock-out should help to forecast demand and therefore make it possible to secure drug availability. These steps can be relatively easily achieved without difficult negotiations between the stakeholders, particularly pharmaceutical companies.

But to stop the outbreak of antimicrobial and vaccine shortages before reaching the level of a pandemic would require: (1) ensuring uninterrupted supply through local manufacturing and appropriate stock management; (2) strengthening the supply chain by ensuring that there are multiple supplies competing at different levels of the production chain, specifically at critical levels. Those two crucial steps call for complete rethinking of the market. To offer a long-term solution, a useful policy would be to offer incentives to manufacturers to reinvest in the antimicrobial drug and vaccine market, to avoid monopoly, to strengthen the supply chain and to actively participate in information exchange with governmental and national/supranational agencies through a global reporting system linking antimicrobial and vaccine consumption, outbreaks and tension over the supply chain.

In addition to this global approach, more specific strategies should be explored. As an example, it has been suggested that military pharmaceutical labs could be authorized to manufacture out-of-stock essential drugs (29). They have the technical capacity to do so, and a license could be accorded for a limited duration until the regular supply chain is available again. That could be both an incentive for pharmaceutical companies to limit and shorten the duration of shortages and a guarantee of undisrupted access to essential drugs.

Fortunately, initiatives toward a global response have been set up, such as European commission call to European health union to manage appropriately crisis such as COVID-19 but also shortages (30) or EMA guidelines to strengthen global approach to development of new antibacterial medicines (31). In 2012, the GAIN Act which provided incentives such as extension of 5-year non-patent exclusivity for some antimicrobial in the USA turned out to be poorly efficient if not counter-productive, but resulted in a better understanding of the market and potentially useful proposals (32).

## Conclusion

Antimicrobial drug and vaccine shortages impact society far beyond the individual level, with unmatched consequences. Resulting from multiple complex issues, our societies are called upon to face considerable challenges, which require a courageous multi-level response involving all stakeholders. A first step toward the solution is to extensively document shortages and their impact in view of raising awareness and motivating governments, agencies and pharmaceutical companies. As a society we can invent new mechanisms and new incentives to make the market attractive (which implies to collectively paying slightly more than we are used to, though maintaining affordability). The other option is to maintain the status quo, which will soon lead the few players remaining in the market to drive prices up without either control or affordability for many countries, with the threat of a new pandemic on the horizon.

## Data Availability Statement

The original contributions presented in the study are included in the article/Supplementary Material, further inquiries can be directed to the corresponding author/s.

## Author Contributions

GB performed literature review, wrote the whole manuscript and edited it himself when requested.

## Conflict of Interest

The author declares that the research was conducted in the absence of any commercial or financial relationships that could be construed as a potential conflict of interest.
